# Disruption of the Lipid-Transporting LdMT-LdRos3 Complex in *Leishmania donovani* Affects Membrane Lipid Asymmetry but Not Host Cell Invasion

**DOI:** 10.1371/journal.pone.0012443

**Published:** 2010-08-26

**Authors:** Adrien Weingärtner, Björn Drobot, Andreas Herrmann, María P. Sánchez-Cañete, Francisco Gamarro, Santiago Castanys, Thomas Günther Pomorski

**Affiliations:** 1 Institute of Biology and Biophysics, Humboldt-University Berlin, Berlin, Germany; 2 Instituto de Parasitología y Biomedicina ‘López-Neyra’, Consejo Superior de Investigaciones Científicas (CSIC), Armilla, Granada, Spain; 3 Department of Plant Biology and Biotechnology, University of Copenhagen, Copenhagen, Denmark; Federal University of São Paulo, Brazil

## Abstract

Maintenance and regulation of the asymmetric lipid distribution across eukaryotic plasma membranes is governed by the concerted action of specific membrane proteins controlling lipid movement across the bilayer. Here, we show that the miltefosine transporter (LdMT), a member of the P4-ATPase subfamily in *Leishmania donovani*, and the Cdc50-like protein LdRos3 form a stable complex that plays an essential role in maintaining phospholipid asymmetry in the parasite plasma membrane. Loss of either LdMT or LdRos3 abolishes ATP-dependent transport of NBD-labelled phosphatidylethanolamine (PE) and phosphatidylcholine from the outer to the inner plasma membrane leaflet and results in an increased cell surface exposure of endogenous PE. We also find that promastigotes of *L. donovani* lack any detectable amount of phosphatidylserine (PS) but retain their infectivity in THP-1-derived macrophages. Likewise, infectivity was unchanged for parasites without LdMT-LdRos3 complexes. We conclude that exposure of PS and PE to the exoplasmic leaflet is not crucial for the infectivity of *L. donovani* promastigotes.

## Introduction

Most eukaryotic cells exhibit an asymmetric distribution of phospholipids in their plasma membrane, with aminophospholipids phosphatidylserine (PS) and phosphatidylethanolamine (PE) restricted to the cytosolic leaflet [1]. This asymmetric lipid distribution is maintained by ATP-dependent processes, suggesting that it is critical to normal cell function. Indeed, if cells fail to engage mechanisms to maintain lipid asymmetry, PS and PE appear at the cell surface, which leads to dramatic changes in cell function [2]. In multicellular organisms, one of the important consequences of altered membrane lipid asymmetry is the recognition and engulfment of PS exposing apoptotic cells by mononuclear macrophages. Recently, PS exposure has also been implicated in the infectivity of *Leishmania*, an obligate, intracellular parasite that infects cells of the mononuclear phagocyte lineage in their vertebrate hosts [3]–[5]. The parasite has a digenic life cycle, residing as flagellated extracellular promastigotes in the gut of the insect vector and as obligatory intracellular aflagellated amastigotes found in the parasitophorous vacuoles of mammalian macrophages. The observation of PS exposure on the cell surface of *Leishmania* parasites led to the hypothesis that the protozoan parasite might take advantage of a regulated loss of lipid asymmetry and PS presentation to invade and survive in host macrophages [6]. However, it remains open whether (i) PS exposure is mandatory for invasion of host cells and (ii) exposure of other lipids may facilitate infectivity as well. In fact, exposure of PS is typically accompanied by a loss of transbilayer asymmetry of other lipids. In particular, PE redistributes to the exoplasmic leaflet as well. Hence, this lipid may contribute to or even be critical for infectivity of parasites.

Maintenance and regulation of membrane lipid asymmetry is governed by the concerted action of ATP-driven translocases that move specific lipids against a concentration gradient across the bilayer. Prime candidates for outward-directed lipid translocases are members of the ATP binding cassette (ABC) transporter family [7], whereas potential inward-directed lipid translocases belong to the P4 subfamily of P-type ATPases [8]. Members of both transporter families have also been implicated in the development of drug resistance in *Leishmania*, which includes resistance to alkyl-lysophospholipids [9]–[14]. This suggests that the mechanism by which the drugs are extruded from cells is closely related to the flippase mechanism by which lipids are translocated across membranes, and that these processes involve structurally similar, if not identical transporters.

Studies aimed at identifying the molecular basis of miltefosine (hexadecylphosphocholine) resistance led to the identification of two *Leishmania* membrane proteins, LdMT, a member of the P4 subfamily of P-type ATPases, and LdRos3, a potential noncatalytic subunit of LdMT related to Cdc50 family [11], [12], [14]. Both proteins are primarily localized to the *Leishmania* plasma membrane and required for the rapid intracellular uptake of alkylphosphocholine drugs. In the budding yeast *Saccharomyces cerevisiae*, members of the two protein families have been found to form stable transporter complexes that function in the translocation of phospholipids from the exoplasmic to the cytoplasmic leaflet of cellular membranes [15], [16]. Likewise, LdMT and LdRos3 have been implicated in the uptake of fluorescent 7-nitrobenz-2-oxa-1,3-diazol-4-yl (NBD)-labelled phospholipids, suggesting a role for these proteins in controlling lipid asymmetry in the parasite plasma membrane [12], [17]. However, lipid asymmetry of parasite plasma membranes is not well defined and the proteins involved in its regulation remain to be identified.

In this work, we study the regulation of phospholipid asymmetry in promastigotes of *Leishmania donovani* and the relevance of asymmetry loss for infection. In particular, we show that LdMT and LdRos3 form a stable protein complex that is essential for inward translocation of NBD-PE and -PC across the parasite plasma membrane. Loss of either LdMT or LdRos3 leads to an increased cell surface exposure of endogenous PE. We therefore propose that the LdMT-LdRos3 complex plays an essential role in maintaining phospholipid asymmetry in the parasites plasma membrane. Since, to our surprise, promastigotes of *Leishmania donovani* lack any detectable amount of PS, we could study specifically the role of PS and PE exposure in host cell invasion. Control cells with functional LdMT-LdRos3 complexes were able to invade host cells showing that PS is not mandatory for infection. Furthermore, infection was not enhanced for parasites without LdMT-LdRos3 complexes indicating that redistribution of PE to the exoplasmic leaflet does not facilitate invasion.

## Results

### Leishmania LdMT and LdRos3 form a stable complex

**Figure 1:**
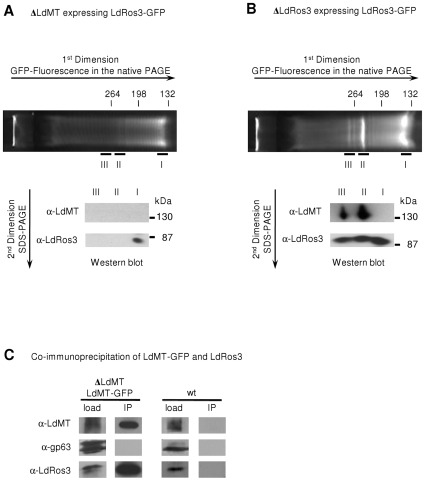
LdMT and LdRos3 form a stable complex in the *Leishmania* membrane. (A, B) Native- and SDS-PAGE analysis of LdMT and LdRos3-GFP. LdRos3-GFP was expressed in ΔLdMT parasites (A) and in ΔLdRos3 parasites (B). Solubilised membrane proteins were separated by native PAGE and analyzed for GFP fluorescence. The membrane extract obtained from ΔLdRos3 parasites contains a prominent fast-migrating fluorescent band (band I). The extract obtained from ΔLdMT parasites also contains slow-migrating fluorescent protein bands (band II and III). Regions of the gel corresponding to the fluorescent bands were excised and loaded onto SDS-PAGE gels which were subsequently immunoblotted with polyclonal antibodies against LdRos3 (α-LdRos3) and LdMT (α-LdMT). Size markers indicate relative mobility of proteins in kDa. (C) Immunoblots from co-immunoprecipitation assays. LdMT-GFP was immunoprecipated from a detergent-solubilised membrane fraction (load) obtained from ΔLdMT parasites expressing LdMT-GFP as well as non-transfected wild-type parasites (wt) using anti-GFP-MicroBeads. Immunoprecipitates (IP) were subjected to immunoblot analysis using antibodies that recognize LdMT (α-LdMT), LdRos3 (α-LdRos3) and metalloprotease gp63 (α-gp63).

To investigate the ability of LdMT and LdRos3 to form a stable complex as their homologues in yeast, a C-terminally green fluorescent protein (GFP)-tagged version of LdRos3 (LdRos3-GFP) was expressed in parasites lacking either LdRos3 (ΔLdRos3) or LdMT (ΔLdMT). Tagged LdRos3-GFP was functional, since it could suppress resistance of ΔLdRos3 parasites to the alkyl-phospholipid derivate miltefosine [12]. Total membrane preparations from the LdRos3-GFP expressing parasites were subjected to solubilisation in the presence of mild detergent and analysed by non-denaturing polyacrylamide gel electrophoresis (native-PAGE), in which complex formation between two proteins gives a new band with mobility different from that of either protein alone. Fluorimaging of the native gels identified one prominent fluorescent band of low molecular size for protein preparations obtained from ΔLdMT parasites expressing LdRos3-GFP (Fig. 1A). In protein samples obtained from ΔLdRos3 parasites expressing LdRos3-GFP, additional fluorescent bands of retarded mobility were detected (Fig. 1B, bands II–III). To test for the presence of LdMT in the GFP-labelled complexes, the fluorescent bands were excised from the native gel and subjected to SDS-PAGE followed by western blot analysis. Immunoblotting with antibodies to LdMT and LdRos3 showed that the fluorescent band of low molecular size (band I) only contained LdRos3-GFP while the more slowly migrating bands (band II-III) corresponded to a complex of LdMT and Ros3-GFP.

To corroborate that LdMT and LdRos3 form a stable complex, a C-terminally GFP-tagged version of LdMT (LdMT-GFP) was introduced into ΔLdMT parasites, and co-immunoprecipitation experiments were performed using anti-GFP-MicroBeads. Tagged LdMT-GFP has been shown to be functional, since it can rescue the miltefosin uptake defect in ΔLdMT parasites [12]. Non-transfected wild-type parasites served as a control. As shown in Figure 1C, the anti-GFP antibodies efficiently precipitated LdMT-GFP and LdRos3 from detergent-solubilised membrane preparations obtained from parasites expressing LdMT-GFP. In contrast, an unrelated integral plasma membrane protein (metalloprotease gp63) was not present in the immunoprecipitate. As a control, a parallel immunoprecipitation from an extract obtained from a wild-type strain lacking the LdMT-GFP fusion did not precipitate LdRos3, indicating that the co-immunoprecipitation was specific. Analysis of the LdMT-GFP immunoprecipitates by mass spectrometry revealed that the preparations did not contain other Cdc50 members than LdRos3 (data not shown). Taken together, these results provide direct evidence that LdMT and LdRos3 reside in a stable complex in the membrane. We infer that the stoichiometry of both proteins in the complex is one because we did not detect corresponding fluorescent bands of retarded mobility for larger complexes.

### LdMT and LdRos3 are required for the ATP-dependent inward translocation of NBD-PC and -PE

**Figure 2:**
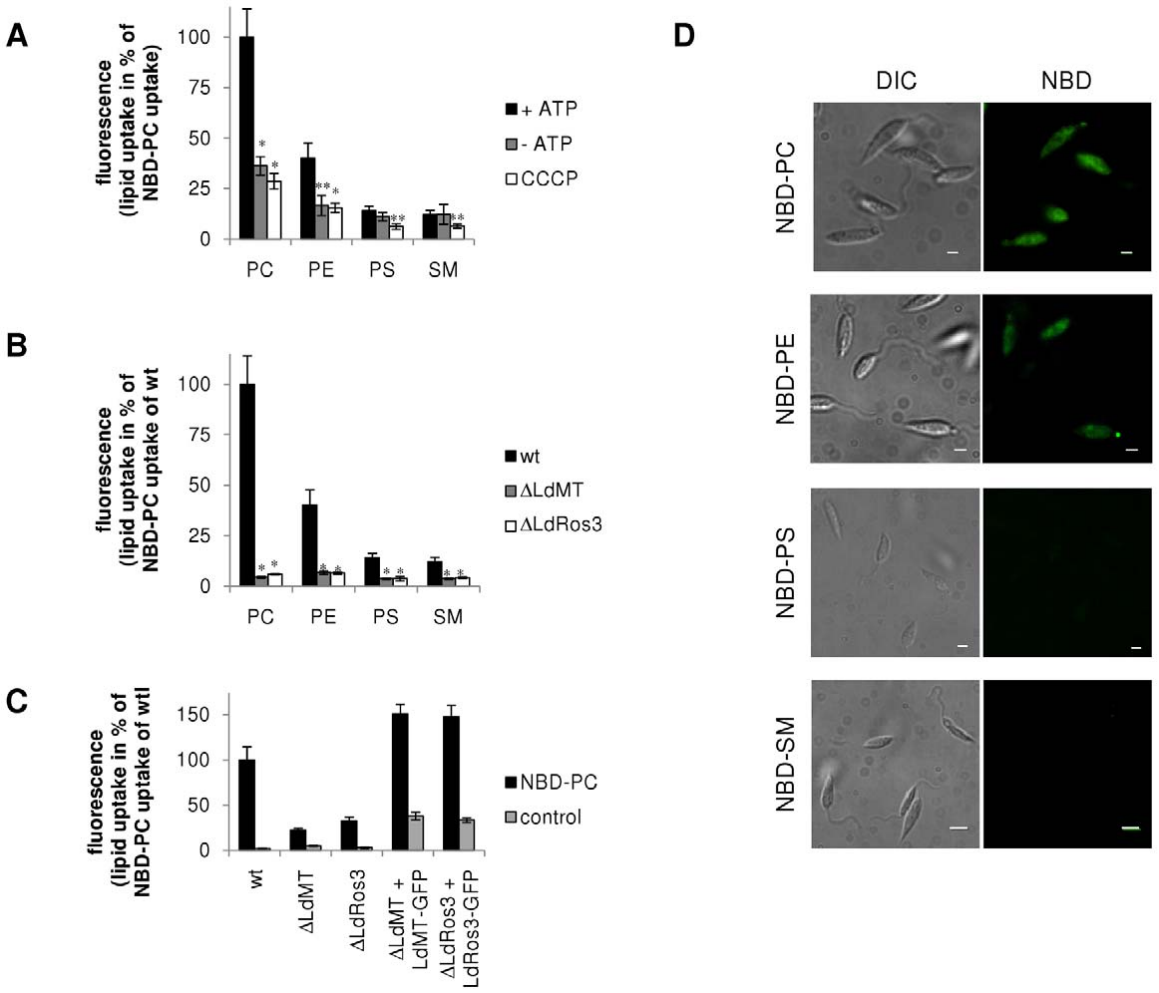
Inward translocation of NBD-PC and NBD-PE across the plasma membrane of *Leishmania* requires LdMT and LdRos3. Promastigotes of wild type (wt), ΔLdMT and ΔLdRos3 lines were labelled with NBD-lipids for 30 min at 2°C and than washed and analysed by flow cytometry (A–C) or visualised by fluorescence microscopy (D). ATP depletion was achieved by preincubation with 5 mM 2-deoxyglucose and 20 mM sodium azide. To abolish the proton electrochemical gradient 50 µM of the protonophore CCCP was used. As a control LdMT-GFP and LdRos3-GFP on episomal *Leishmania* expression vectors were reintroduced in ΔLdMT and ΔLdRos3 mutants, respectively; control, non-labelled cells showing the intrinsic fluorescence of the GFP fusion proteins. Data are normalized to NBD-PC internalization of wild-type parasites; 100% corresponds to 468±96 a.u. NBD-PC. Data represent the means ± SE of at least three independent experiments. For statistical analysis Welch's test was performed. Significant differences in the lipid uptake of the mutants compared to the wild type are denoted by asterisks (** p = 0.05; * p = 0.01).

To characterize the lipid transport activity of the LdMT-LdRos3 complex in the plasma membrane we examined in wild type, ΔLdMT and ΔLdRos3 parasites the uptake of NBD-lipids. This can be monitored by flow cytometry and by fluorescence microscopy. Experiments were performed at 2°C to suppress endocytosis and suppress degradation of the NBD-lipids (consistently <10%). Under these conditions, wild-type parasites efficiently internalized NBD-PC and NBD-PE, while NBD-PS and NBD-sphingomyelin (NBD-SM) were hardly taken up at all. The efficient uptake of NBD-PC and NBD-PE was significantly inhibited following ATP depletion by preincubation with sodium azide and 2-deoxyglucose or collapse of the proton electrochemical gradient with the protonophore carbonyl cyanide m-chlorophenylhydrazone (CCCP) (Fig. 2A). Consistent with these results fluorescence microscopy of wild-type parasites labelled with NBD-lipids revealed an intensive labelling of intracellular membranes with NBD-PC and -PE but not with NBD-PS and -SM (Fig. 2D). In contrast to wild-type parasites, ΔLdMT and ΔLdRos3 parasites were found defective in the low temperature uptake of NBD-PC and NBD-PE (Fig. 2B). These defects were due solely to the loss of LdMT and LdRos3, as lipid uptake was restored by re-expression of LdMT-GFP and LdRos3-GFP in ΔLdMT and ΔLdRos3 mutants, respectively (Fig. 2C). Based on these findings, we conclude that the LdMT-LdRos3 complex is essential to sustain an energy-dependent influx of NBD-PC and -PE across the parasite plasma membrane.

### LdMT and LdRos3 sustain plasma membrane PE asymmetry in *L. donovani*

**Figure 3:**
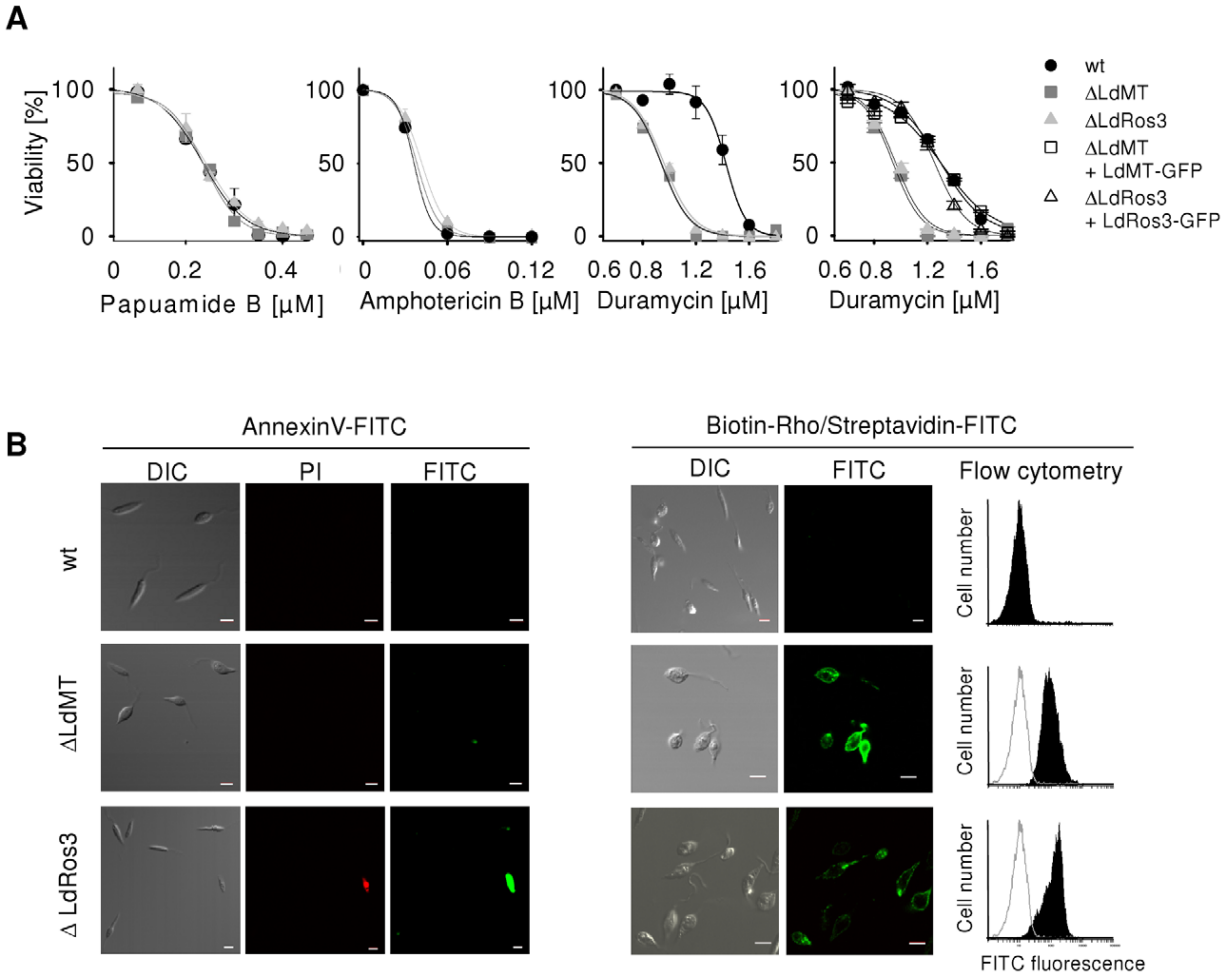
LdMT and LdRos3 are required to sustain plasma membrane PE asymmetry in *Leishmania*. (A) Sensitivity of wild type (wt), ΔLdMT and ΔLdRos3 *L. donovani* parasites to the PE-binding peptide duramycin, the PS-binding peptide papuamide B and the sterol-binding amphotericin B. Parasites were diluted to 0.25×10^6^ cells/ml in medium containing duramycin, papuamide B or amphotericin B at the indicated concentrations. 72 h later viability was analysed as described in the Material and methods. As a control LdMT-GFP and LdRos3-GFP on episomal *Leishmania* expression vectors was reintroduced in ΔLdMT and ΔLdRos3 mutants, respectively. Means ± S.E. of at least three independent experiments are shown as percentage of untreated control parasites. (B) Endogenous PS and PE at the exoplasmic leaflet of the plasma membrane of these strains was visualised with annexin V-FITC and Biotin-Rho/Streptavidin-FITC, respectively. Cells were analyzed by phase contrast (DIC) and fluorescence microscopy (PI, FITC). Bar, 10 µm. Fluorescence intensity histograms were obtained by flow cytometry as described under “Materials and Methods.” Cells incubated in the absence of biotinylated Ro09-0198 peptide served as controls (dash line).

We next tested whether the LdMT-LdRos3 complex helps to maintain a tight asymmetric distribution of aminophospholipids at the plasma membrane. Promastigote stages of *Leishmania* wild type, ΔLdMT and ΔLdRos3 lines were incubated with different concentrations of duramycin and papuamide B, cytolytic peptides that requires binding to cell surface-exposed PE and PS, respectively, to exert their cytotoxicity [18]. As shown in Figure 3A, both ΔLdMT and ΔLdRos3 lines were more sensitive to duramycin-induced cytolysis as compared to the wild-type line with an EC50 of duramycin that was approximately 1.8-fold lower than that for wild-type cells. Restoration of LdMT and Ros3 expression returned the duramycin sensitivity profile back to the wild-type pattern (Figure 3A). There was no significant difference between the ΔLdMT and the ΔLdRos3 *Leishmania* lines as compared to wild-type cells with respect to their sensitivity to papuamide B and amphotericin B, a polyene macrolide antibiotic that binds to membrane ergosterol and induces cellular leakage [19]. To more directly visualize that deletion of LdMT or LdRos3 affects the lipid asymmetry in the plasma membrane, we analyzed the exposure of PE by labelling with biotinylated Ro09-0198, a peptide that specifically binds to PE [20], [21]. As shown in Figure 3B, both the ΔLdMT and the ΔLdRos3 *Leishmania* lines bound more PE-sensing biotinylated Ro09-0198 peptide which was visualized with Streptavidin-FITC. For a quantitative assessment of Ro09-0198 peptide binding, the FITC fluorescence associated with mutant and wild-type cells was measured by flow cytometry. As shown in Figure 3A, deletion of LdMT or LdRos3 caused a 10-fold increase in PE exposure when compared to wild type parasites based on Ro09-0198 peptide binding. We could, however, not detect in any of the lines labelling of propidium iodide (PI) negative parasites with annexin V-FITC, a protein that preferentially interacts with membranes containing PS [22].

**Figure 4:**
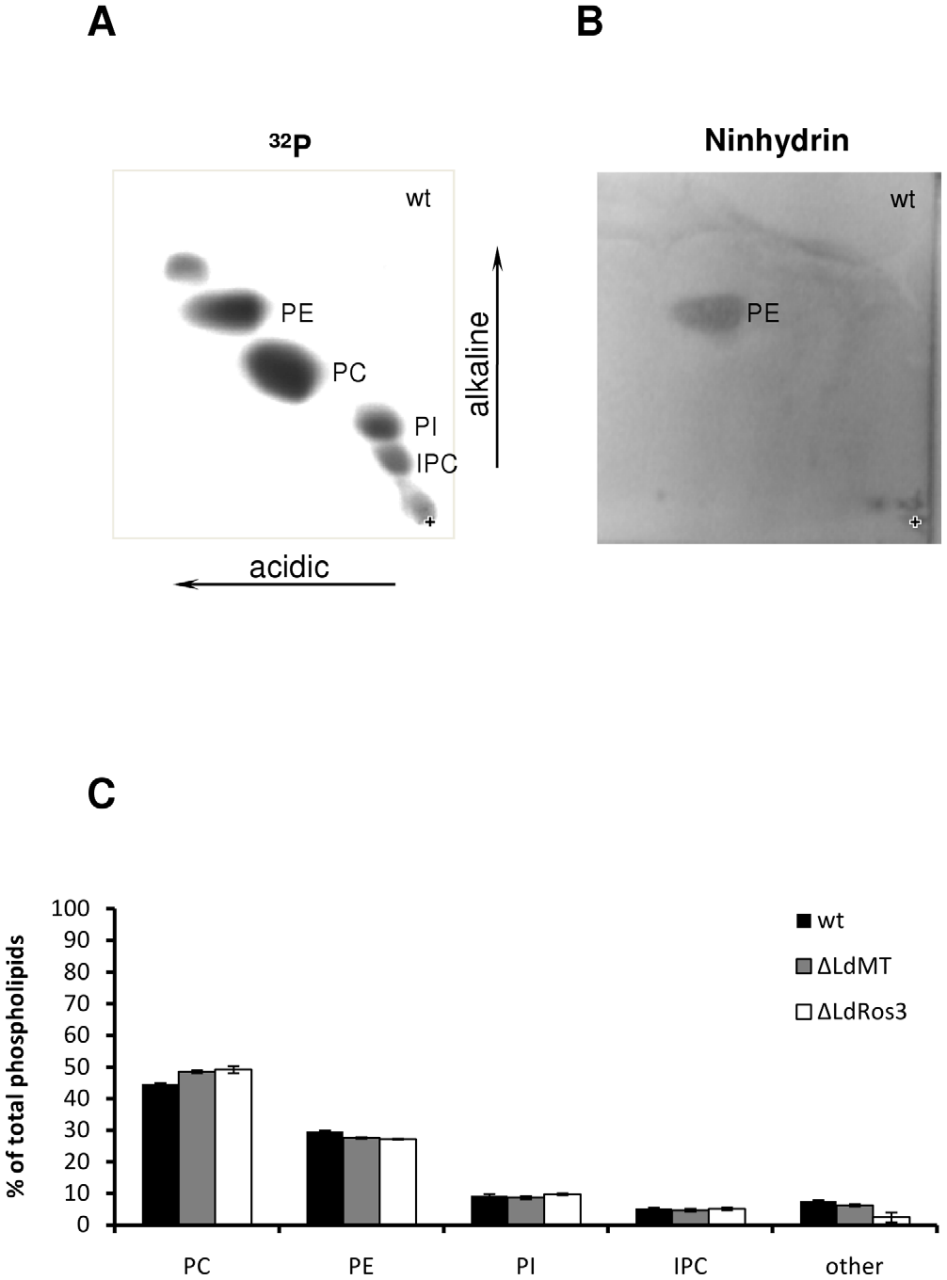
Total phospholipid composition of wild type, ΔLdMT and ΔLdRos3 *L. donovani* parasites. Promastigote stages of wild type (wt), ΔLdMT and ΔLdRos3 lines were labelled for 48 h with ^32^P-phosphate. Lipids were extracted, separated by two-dimensional thin layer chromatography, and then visualized by phosphorimager scanning (A) or ninhydrin staining (B). Representative two-dimensional TLC plates are shown. The location of individual species was verified by ESI-MS. Unidentified lipids are not marked. (C) Quantification of phospholipids in wild-type, ΔLdMT and ΔLdRos3 parasites. Data are expressed as the percentage of total phospholipids and represent the means ± S.E. of three independent experiments. PC, phosphatidylcholine; PE, phosphatidylethanolamine; PI, phosphatidylinositol; IPC, inositolphosphorylceramide.

To rule out the possibility that the observed differences in PE exposure resulted from substantial increase in the PE level in the ΔLdMT and the ΔLdRos3 lines as compared to wild-type parasites, we analyzed the total phospholipid composition in *Leishmania* parasites labelled to steady state with ^32^P-phosphate. Four major phospholipids could be identified with comparable levels in all of the strains: PC (44%–49%), PE (27%–29%), phosphatidylinositol (PI, 9%) and inositolphosphorylceramide (IPC, 4–5%) (Fig. 4A). Thus, the possibility that increased cell surface exposure of PE was caused by increased PE synthesis can be ruled out. Notably, we could not detect significant levels of PS by this method. When stained with ninhydrin, a reagent labelling lipids with free amino groups such as PS and PE, the chromatograms exhibited only one spot corresponding to PE (Fig. 4B). Likewise, lipid analysis by HPLC coupled ESI-MS did not reveal the presence of PS, suggesting that *L. donovani* does, if it all, synthesize very low amounts of this phospholipid (Fig. 4C). The presence of only very low amounts of PS explains the equal sensitivity of the parasite lines to papuamide B and the lack of FITC-annexin V binding on intact parasites (see above).

### Phagocytosis of ΔLdMT and ΔLdRos3 *Leishmania* lines in THP-1-derived macrophages is unchanged

**Figure 5:**
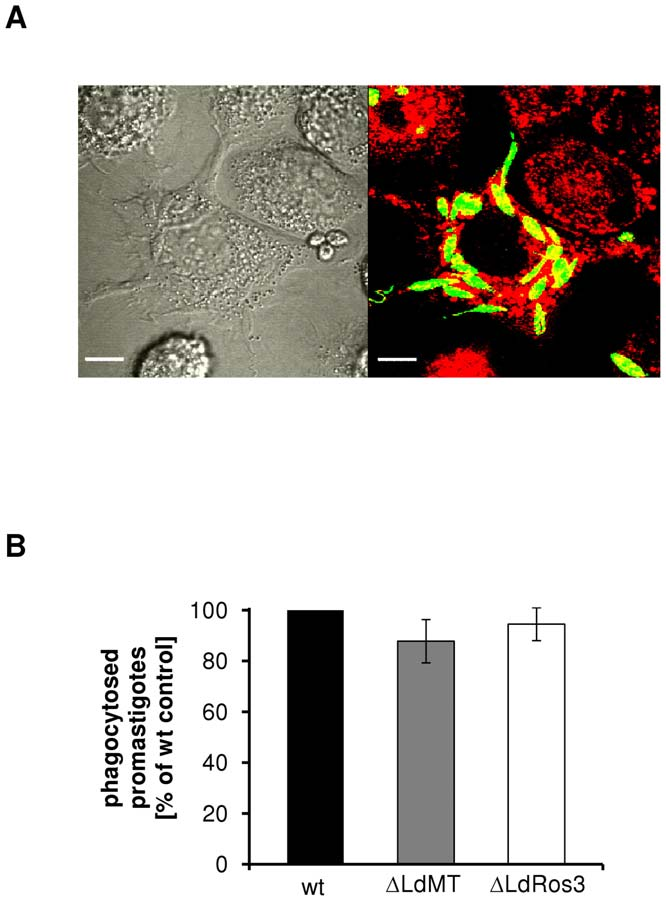
Phagocytosis of ΔLdMT and ΔLdRos3 parasites by THP-1-derived macrophages is unchanged. CellTrackerTM Green-labelled parasites were added (10∶1) to THP-1-derived macrophages prelabelled with CellTrackerTM Dil (red) and co-incubated for 16 h at 37°C. (A) Micrograph of double-fluorescence labelling of THP-1-derived macrophages phagocytosing *Leishmaina* parasites. Bar, 10 µm. (B) Percentage of phagocytosing THP-1-derived macrophages was determined by flow cytometry. Values are means ± SD of three independent experiments expressed as percentage of control (number of phagocytosed wild-type parasites by TPH-1-derived macrophages). The population of phagocytosing THP-1-derived macrophages ranged from 30 to 70% in control cultures.

Exposure of PS on the exoplasmic leaflet of the *Leishmania* plasma membrane has been implicated in entry of the parasite into host cells [3]–[5]. The availability of two *Leishmania* lines strains displaying an altered plasma membrane lipid asymmetry and apparent lack of PS provided an opportunity to test whether other phospholipids are relevant for the recognition of *Leishmania* parasites by macrophages using the human monocytic cell line THP1. THP1 cells have many characteristics of human monocytes, including morphology, surface-membrane receptor, and the capacity to undergo maturational changes when induced with phorbol esters [23], [24]. We used early log phase promastigotes to evade apoptotic subpopulations which increase during late logarithmic and stationary phase. Parasites and THP-1-derived macrophages were prelabelled with CellTrackerTM Green and CellTrackerTM Dil, respectively, and co-incubated at a ratio of 1∶10 (cells∶parasitetes). 16 h post infection, 30 to 70% of the THP-1-derived macrophages were found to be infected. At this moment the number of intracellular parasites per infected macrophage ranged from 1 to 19 (Fig. 5A). The mean numbers of intracellular parasites per infected cell were similar: 2.6 for wt, 2.5 for ΔLdMT and 2.7 for ΔLdRos3 parasites, respectively. The total numbers of THP-1-derived macrophages infected by ΔLdMT and ΔLdRos3 parasites were not markedly different from that of wild-type parasites (Fig. 5B), indicating that at least in this infection model, changes in the plasma membrane PE arrangement of *Leishmania* parasites do not trigger specific recognition and removal of the parasites by macrophages.

## Discussion

Previous studies revealed a critical role for both the miltefosine transporter LdMT and the LdRos3 protein in the uptake and potency of alkylphosphocholine type drugs and in phospholipid translocation at the plasma membrane of *Leishmania* parasites [11], [12], [14]. The present study provides direct evidence that both proteins form a stable complex that is essential for maintaining the normal asymmetric lipid distribution of at the parasite plasma membrane. Loss of plasma membrane PE asymmetry by disruption of the LdMT-LdRos3 complex does not alter the infectivity in macrophages. We conclude that in *L. donovani* loss of the normal asymmetric lipid distribution does not facilitate host cell invasion.

Formation of a stable complex between P4-ATPases and Cdc50 family members seems to be indispensable for a proper targeting and functioning of P4-ATPases. In yeast, the Cdc50 family members Cdc50p, Lem3p and Crf1p can be co-immunoprecipitated with Drs2p, Dnf1p/Dnf2p and Dnf3p, respectively. Formation of these complexes is required for proper expression and endoplasmic reticulum (ER) export of either partner [15], [16]. The human P4-ATPase ATP8B1 requires the Cdc50 member CDC50A, for ER exit and delivery to the plasma membrane [25], and the *Arabidopsis* P4-ATPase ALA3 requires its Cdc50 binding partner ALIS1 to complement the lipid transport defect at the plasma membrane in *Δdrs2Δdnf1Δdnf2* yeast mutant [26]. These findings hold also true for LdRos3 and the P4-ATPase LdMT. However, a closer inspection of the partially sequenced genome of *L. infantum* (http://www.genedb.org/genedb/linfantum/) reveals in total five genes encoding P4-ATPases (LinJ09_V3.0940, LinJ13_V3.1590, LinJ30_V3.2270, LinJ30_V3.2270, LinJ34_V3.2460, LinJ34_V3.3000) and three genes encoding Cdc50 family members (LinJ09_V3.1080, LinJ32_V3.0540, LinJ35_V3.3450). Coming challenges are to identify whether all of these P4-ATPase require a Cdc50 binding partner for proper functioning and whether they can interact with several Cdc50 proteins as shown for the *Arabidopsis* P4-ATPase ALA3 [26]. In light of the results presented in this study, it seems that *Leishmania* LdMT does not associate with other Cdc50 members than LdRos3 and that the stoichiometry of both proteins in the complex is one.

Incubation of parasites with fluorescent phospholipid analogues (PC, PE, PS and SM) served as an approach to analyze lipid transport activity of the LdMT-LdRos3 complex at the plasma membrane. We found that *L. donovani* parasites efficiently internalize NBD-PC and NBD-PE even at 2°C while NBD-PS and NBD-SM are hardly taken at this temperature. Since endocytosis is blocked under this condition [27], uptake of NBD-PC and NBD-PE must involve a translocation step across the plasma membrane. Loss of either LdMT or LdRos3 completely blocked the ATP-dependent uptake of NBD-PC and NBD-PE, indicating that the LdMT-LdRos3 complex facilitates the energy-coupled transport of natural PE and PC from the outer to the inner leaflet of the parasites plasma membrane. In support of this notion, loss of either LdMT or LdRos3 led to an increased cell surface exposure of natural PE, as evidenced by enhanced hypersensitivity to PE-binding peptide and labelling by biotinylated PE-binding peptide. These results are in line with a direct role of the LdMT-LdRos3 complex in pumping natural PE to the cytosolic leaflet to generate an asymmetric membrane as recently suggested for P4-ATPases from other organisms [28], [29]. The ability of wild-type parasites to take up NBD-PC suggests that natural PC is restricted to the inner leaflet of the parasites plasma membrane and that the outer leaflet is primarily composed of sphingolipids, as NBD-sphingomyelin is not taken up.

The substrate specificity of the inward translocation machinery appears to vary between *Leishmania* species. In contrast to *L. donovani*, *L. infantum* displays not only an ATP-dependent internalization of NBD-PC and NBD-PE but also an active transport of NBD-PS across its plasma membrane [30]. Thus, this parasite might express one or more P4-ATPases of broader or different substrate specificity at the plasma membrane, respectively. The physiological relevance of these species-specific differences in substrate specificity remains to be established. Previous studies suggested that the LdMT-LdRos3 complex of *L.donovani* also recognizes NBD-PS, however, as a poor substrate [11], [12]. Significant uptake of NBD-PS in this species was only observed when higher label concentrations were used as compared to NBD-PC and NBD-PE. It is noteworthy that in agreement with former reports [31], [32] we could not detect endogenous PS in total lipid extracts prepared from *L. donovani* promastigotes, suggesting that this parasite does not synthesise considerable amounts of PS. Thus, an inward-directed transport activity for PS at the plasma membrane might not be required in *L. donovani*. However, all experiments undertaken in the present work were performed on the promastigote early stage. It will be interesting to determine whether *L. donovani* amastigotes synthesise PS and regulate its distribution in the parasite plasma membrane. A stage specific regulation of the plasma membrane asymmetry is conceivable since amastigotes up-regulate the expression of two P4-ATPases (LmjF30.2260, LmjF34.3220) and a plasma membrane ABC transporter associated with lipid export (LmjF11.1260) [33]. *Leishmania* amastigotes replicate within the mature phagolysosome compartment and have complex nutritional requirements which must be scavenged from the host cell [34]. It is therefore tempting to speculate that the LdMT-LdRos3 transporter complex or related P4-ATPases are involved in the acquisition of host phospholipids.

The surface of the *Leishmania* parasite is a major point of interaction with the host throughout the infectious cycle. A number of surface glycoconjugates such as lipophosphoglycans, glycosylphosphatidylinositol (GPI)-anchored proteins (e.g. the metalloprotease gp63), and a heterogeneous group of small glycosylinositolphospho-lipids have been implicated in the ability of the parasite to infect and survive in host macrophages [35]. In addition, surface exposed PS has been associated with parasite infectivity [3]–[5]. However, these results must be interpreted with caution because PS exposure was concluded on basis of annexin V and anti-PS antibody binding in these studies. Despite being used extensively to label PS, neither annexin V nor anti-PS antibodies are specific for this lipid and also bind phosphatidic acid, phosphatidylglycerol and phosphatidylinositol-4,5-bisphosphate [36]. Thus, a direct proof for PS exposure by *Leishmania* parasites is currently lacking. In fact, we observed that *L. donovani* promastigotes lack significant amounts of PS, and annexin V-FITC does not label living, PI negative *L. donovani* parasites but parasites positive for PI staining. Conceivably, the various *Leishmania* spp. might expose other lipids than PS that contribute to the infectivity. Our results on ΔLdMT and the ΔLdRos3 *Leishmania* mutant lines that display an altered PE asymmetry did not reveal major differences in terms of invasion efficiency into the human monocytic cell line THP-1. In line with these results, the ΔLdMT mutant remains infective and maintains virulence in cultures of primary isolated mouse peritoneal macrophages [37]. These results suggest that disruption of the LdMT-LdRos3 transporter complex and changes in the transbilayer distribution of PE, and probably PC, are not crucial for the infectivity of *L. donovani* promastigotes. Likewise, in the THP-1 infection model used here PS exposure is not mandatory for invasion of host cells. Future studies on other *Leishmania* species and mutants lacking the ability to synthesized PS may help to define in more detail the lipid types exposed on the cells surface and involved in parasite internalization and phagocyte inactivation.

## Materials and Methods

### Culture and transfection of Leishmania lines

Promastigotes of *L. donovani* (MHOM/ET/67/HU3) and derivative lines LdMT knockout (ΔLdMT) and LdRos3 knockout (ΔLdRos3) [12] were maintained at 26°C in M-199 medium (Invitrogen, Karlsruhe, Germany) supplemented with 40 mM HEPES, 100 µM adenosine, 0.5 µg/ml hemin, 10 µM 6-biopterin and 10% heat inactivated fetal calf serum (Gibco, Invitrogen GmbH, Karlsruhe, Germany). Parasites (3×10^7^ promastigotes) were transfected with the C-terminal fusion constructs LdMT-GFP or LdRos3-GFP by electroporation (450 V, 500 µF) and then maintained in culture medium with 200 µg/ml G418 [11]. Unless indicated otherwise, all materials were purchase from Sigma-Aldrich (Taufkirchen, Germany).

### Protein analysis and immunoprecipitation

For preparation of membrane proteins, parasites were harvested, resuspended in ice-cold hypo-osmotic lysis buffer (5 mM Tris-HCl pH 7.4) containing 1 mM phenylmethylsulfonyl fluoride, and then broken by vortexing with glass beads. The cell lysate was clarified by centrifugation at 500×g (10 min, 4°C). Subcellular membranes were collected by centrifugation at 100,000×g (1 h, 4°C) and resuspended in sample buffer (750 mM 6-aminocarproic acid, 50 mM Bistris pH 7.0, 20% glycerol) to a protein concentration of 5 mg/ml. Protein concentration was measured using the BCA protein assay kit (Pierce Chemical Company, Rockford IL, USA). Membranes were solubilised by adding 20 µl of n-dodecyl-β-D-maltopyranoside (DDM, 10%) to 100 µl suspended membranes, corresponding to a DDM/protein ratio of 4 (w/w). After incubation for 60 min on ice, insoluble material was removed by centrifugation (100,000×g, 1 h, 4°C).

For Clear Native-PAGE, solubilised membrane proteins (1 mg) were loaded directly onto a 6%–15% gradient gel (6%–15% acrylamid/bisacrylamid in 50 mM Bis Tris-HCl pH 7.0, 500 mM 6-aminocarproic acid). Electrophoresis was carried out at 4°C at 500 V for 16 h (electrophoresis buffer  = 50 mM Tricine, 15 mM Bis Tris-HCl pH 7.0, 0.05% Na-taurodeoxycholat, pH 7.0) and bovine serum albumin (monomer, 66 kDa; dimer, 132 kDa; trimer, 198 kDa; tetramer, 264 kDa) was used as molecular weight marker. Gels were scanned for GFP fluorescence using a Fuji FLA3000 phosphoimager (Raytest GmbH, Straubenhardt, Germany) equipped with a 473 nm argon laser and 510 nm long pass filter. Fluorescent bands were excised from the native gel and subjected to SDS-PAGE followed by western blot analysis.

GFP-co-immunoprecipitation assays were performed using anti-GFP microBeads (Miltenyi Biotec, Bergisch Gladbach, Germany). Per immuno-isolation, a 1 ml reaction was prepared in lysis buffer containing 150 µl of anti-GFP microBeads slurry, 250 µl of detergent-solubilised protein. The reactions were rotated gently at 4°C for 30 min. Beads were separated from the supernatants using µColumns with a µMACS separator (Miltenyi Biotec) and washed three times with lysis buffer containing 0.05% DDM. Membrane protein extracts and immuno-isolated membrane proteins were subjected to western blot analysis. Immunoblots were probed with polyclonal antibodies against LdMT and LdRos3 [14], and gp63 (kindly provided by Robert McMaster). Horseradish peroxidase-conjugated secondary antibodies were from Bio-Rad (Hercules, CA). Blots were developed using enhance chemiluminescence (ECL plus Kit, GE-Healthcare, Freiburg, Germany). Protein mass spectrometry of the immunoprecipitates was performed as described elsewhere [38].

### NBD-lipid uptake and flow cytometry analysis in *L. donovani* lines

Palmitoyl-(NBD-hexanoyl)-phosphatidylserine (NBD-PS), -phosphatidylethanolamine (NBD-PE), -phosphatidylcholine (NBD-PC) and 6-NBD-hexanoyl-sphingosine-1-phosphocholine (NBD-sphingomyelin; NBD-SM) were from Avanti Polar Lipids (Birmingham, AL). Appropriate amounts of analogues (5 nmol of NBD-lipids for 10^7^ cells) in chloroform/methanol (1∶1) were transferred to a glass tube, dried under nitrogen, dissolved in 5 µl absolute ethanol and vortexed with the desired volume of HPMI (132 mM NaCl, 3.5 mM KCl, 0.5 mM MgCl_2_, 1 mM CaCl_2_, 5 mM glucose, 1 mM pyruvate, 20 mM HEPES, pH 7.4). Promastigotes in logarithmic phase of growth were harvested by centrifugation (1000×g, 10 min, 4°C), washed twice with HPMI. To block the catabolism of NBD-lipids the cell suspension (10^7^ parasites/ml) was pre-incubated with 5 µM 3-(4-octadecyl)-benzoylacrylic acid (Biomol, Hamburg, Germany) and 1 mM phenylmethanesulphonylfluoride for 30 min at 28°C. To study the lipid uptake, the parasites were then incubated at 4°C with 5 µM NBD-lipid. After 30 minutes, cells were washed twice in HPMI containing 4% (w/v) fatty acid free bovine serum albumin to extract NBD lipids from the cell surface. Flow cytometry analysis was performed on a Becton Dickinson FACS (San Jose, CA) equipped with an argon laser (488 nm) using Cell Quest software. One µl of 1 mg/ml PI in H_2_O was added 200 µl cell suspension just before analysis. Ten thousand cells were analyzed at room temperature with gating during the acquisition. Live cells were selected based on forward/side-scatter gating and propidium iodide exclusion. The following fluorescence channels (log scale) were used: FL1 (530/30 nm, FITC, NBD, CellTrackerTM Green), FL2 (585/42 nm, PI, CellTrackerTM Dil). Data were analyzed by Cyflogic software.

### Drug Sensitivity Assays in *L. donovani* lines

To determine parasite sensitivity to amphotericin B, papuamide B (Flintbox, Lynsey Huxham) and duramycin, 2,5×10^4^ parasites were incubated in 96well plates (100 µl) for three days at different drug concentrations before determining cell proliferation by the 3-(4,5-dimethyl-2-thiazolyl)-2,5-diphenyl-2H-tetrazolium bromide (MTT) colorimetric assay as previously described [11]. The 50% effective concentration (EC50) was defined as the drug concentration required for half-maximal inhibition of the cellular growth rate. The EC50 for each line was calculated by nonlinear regression analysis using SigmaPlot 2000 for Windows (SPSS Inc., Chicago, IL, USA).

### Annexin V und Bio-Rho Assay

To visualize endogenous PE on the cell surface, 5×10^6^ promastigotes in logarithmic phase of growth were incubated in 20 µl HPMI containing 38 µM biotinylated Ro09-0198 (provided by Kazuma Tanaka). After 1 h at 4°C, cells were washed with phosphate buffered saline (PBS) containing 0.5% (w/v) bovine serum albumin and then fixed with PBS containing 5% (w/v) formaldehyde for 1 h at 30°C. Promastigotes were then washed in PBS, resuspended in 250 µl PBS containing 5 µg/ml Streptavidin-FITC and incubated for 30 min at 25°C prior to microscopy analysis. To measure exposure of endogenous PS on the cell surface, about 5×10^5^ parasites were incubated on ice for 10 min in the dark with 125 ng annexin V-FITC and 1 µg PI in 0.5 ml of binding buffer (10 mM HEPES pH 7.4, 140 mM NaCl, 2.5 mM CaCl_2_). Cells were washed, resuspended in 0.5 ml of binding buffer and subjected to microscopy.

### Metabolic labelling and lipid fractions analysis

Parasites (5×10^7^) were inoculated in 100 ml of supplemented M-199 containing 100 µCi [^32^P]H_3_PO_4_ (1 mCi, Hartmann-Analytic GmbH, Braunschweig, Germany) and grown for 48 h at 26°C. Cells were harvested by centrifugation, washed twice with PBS. Total cellular lipids were extracted by the method of Bligh and Dyer [39] and separated by two-dimensional thin layer chromatography (I: chloroform/methanol/25% aqueous ammonium hydroxide, 90∶54∶7; II: chloroform/acetone/methanol/acetic acid/water, 50∶20∶10∶10∶5). The ^32^P-containing radiolabelled spots were imaged on a ^32^P-sensitive screen and quantified on a Fuji Imaging System imager (FLA3000, Raytest, Straubenhardt, Germany). Lipids were also visualized with common lipid-locating agents such as iodine or ninhydrin (0.25% ninhydrin w/v in acetone). For lipid determination by HPLC (Agilent 1200 series) coupled to ESI-MS (Finnigan LTQ Fourier transform ion cyclotron resonance) lipid spots from iodine stained 2D-TCL were scraped of, extracted as described above and dried. The HPLC column (BioBasic-4 C4 5 µ 150×1 mm) was rinsed with 70% Buffer B (70% acetonitril, 25% 2-propanol, 5% H_2_O; 10 mM triethylammonium acetat, 1 mM acetic acid) and 30% Buffer A (95% H_2_O, 5% acetonitril; 10 mM triethylammonium acetat, 1 mM acetic acid) first. Next, the lipids (dissolved in acetonitril/methanol (1∶1)) were loaded to the column and the concentration of Buffer A was increased stepwise to 100%. Eluted fractions were directly injected to the ESI-MS and analysed in negative ion mode.

### Phagocytosis of *L. donovani* lines by macrophages

THP-1 cells were cultured at 37°C with 5% CO_2_ in RPMI-1640 medium (Gibco) supplemented with 2 mM glucose, 50 µM β-mercaptoethanol, 1 mM Na-pyruvate, and 10% fetal calf serum. To stimulate differentiation into macrophages, 100 ng/ml phorbol 12-myristate 13-acetate (Biomol, Hamburg) was added to THP-1 cells (2×10^5^/ml). After 12 h, adherent cells were washed 3 times with PBS and cultured as described above. Three days post-differentiation, THP-1 cells (10^6^/flask) were labelled for 40 min in 3 ml RPMI-1640 medium containing 0.5 µM CellTrackerTM Dil (Invitrogen). Non-accumulated dye was removed by washing the cells 5 times with RPMI-1640 medium. Promastigotes (10^7^) from early log phase were labelled for 40 min in 2.5 ml M-199 medium containing 0.5 µM CellTrackerTM Green (Invitrogen). Then, parasites were washed five times with M-199 and finally resuspended in RPMI-1640. To study phagocytosis of promastigotes by macrophages, about 10^7^ CellTrackerTM-labeled parasites were added to 10^6^ CellTrackerTM Dil-labeled adherent macrophages (macrophage∶parasite ratio, 1∶10). After 16 h, cultures were analysed by flow cytometry and fluorescence microscopy. For flow cytometry, macrophages were rinsed intensively with PBS, trypsinised for 20 min at 37°C and finally suspended in supplemented RPMI-1640.

### Fluorescence Microscopy

Epifluorescence microscopy and image acquisition were carried out using an inverse Axiovert 100 standard fluorescence microscope (Carl Zeiss, Oberkochen, Germany) equipped with a cooled CCD camera (Coolsnap, visitron systems, Puchheim, Germany) driven by Metamorph software (Universal Imaging, Downingtown, USA). NBD fluorescence was viewed using a Plan-APO 100×/1.3 NA oil objective with the following filter set: BP 450–490, FT 510, and BP 512–542. Confocal laser scanning microscopy was performed using an inverted Fluoview 1000 microscope (Olympus, Tokio, Japan) and a 60× (N.A. 1.35) oil-immersion objective. Fluorescence of FITC and CellTracker Green was excited with a 488 nm argon laser and recorded between 500 and 530 nm. Fluorescence of PI and CellTracker CM-Dil was excited with a 559 nm argon laser and recorded between 570 and 600 nm. Images with a frame size of 256×256 pixels were acquired.
